# Marker-trait associations and genomic predictions of interspecific pear (*Pyrus*) fruit characteristics

**DOI:** 10.1038/s41598-019-45618-w

**Published:** 2019-06-21

**Authors:** Satish Kumar, Chris Kirk, Cecilia Hong Deng, Angela Shirtliff, Claudia Wiedow, Mengfan Qin, Jun Wu, Lester Brewer

**Affiliations:** 1 The New Zealand Institute for Plant and Food Research Limited, Hawke’s Bay Research Centre, Havelock North, New Zealand; 2 The New Zealand Institute for Plant and Food Research Limited, Palmerston North Research Centre, Palmerston North, New Zealand; 3 The New Zealand Institute for Plant and Food Research Limited, Mount Albert Research Centre, Auckland, New Zealand; 4 Centre of Pear Engineering Technology Research, Nanjing Agricultural University, Nanjing, 210095 China; 5 The New Zealand Institute for Plant and Food Research Limited, Motueka Research Centre, Motueka, New Zealand

**Keywords:** Molecular biology, Plant sciences

## Abstract

Interspecific pear (*Pyrus* spp.) hybrid populations are often used to develop novel cultivars. Pear cultivar breeding is a lengthy process because of long juvenility and the subsequent time required for reliable fruit phenotyping. Molecular techniques such as genome-wide association (GWA) and genomic selection (GS) provide an opportunity to fast-forward the development of high-value cultivars. We evaluated the genetic architecture of 10 pear fruit phenotypes (including sensory traits) and the potential of GS using genotyping-by-sequencing of 550 hybrid seedlings from nine interrelated full-sib families. Results from GWA suggested a complex polygenic nature of all 10 traits as the maximum variance explained by each marker was less than 4% of the phenotypic variance. The effect-size of SNPs for each trait suggested many genes of small effect and few of moderate effect. Some genomic regions associated with pear sensory traits were similar to those reported for apple – possibly a result of high synteny between the apple and pear genomes. The average (across nine families) GS accuracy varied from 0.32 (for crispness) to 0.62 (for sweetness), with an across-trait average of 0.42. Further efforts are needed to develop larger genotype-phenotype datasets in order to predict fruit phenotypes of untested seedlings with sufficient efficiency.

## Introduction

Pear is currently grown commercially in almost every continent of the world. The number of catalogued species in the genus *Pyrus* varies according to different studies, but commercial breeding has mainly focussed on three species: *P*. *communis* (European pear), and two Asian pears namely *P*. *pyrifolia* and *P*. x *bretschneide*ri^1^. High flavour, buttery and juicy texture are among the key characteristics of European pears, while Asian pears generally have crisp texture and subtle flavour. Breeding programmes in New Zealand and elsewhere have combined Asian and European pears to develop crisp, juicy and highly flavoured hybrid cultivars.

Pear breeding programmes generally involve mating of selected parents to create hybrid seedling populations, selection amongst seedling populations, and testing of best performing seedlings. Traditional pear breeding is an expensive and lengthy process primarily because seedlings grown on their own roots typically have a long juvenile period. Reduction of generation time is a focus of many breeding programmes as this has the largest influence on the time taken for new products to reach market^2^. One way of reducing the juvenility period is by growing seedlings in the glasshouse to accelerate the growth rate before planting them into the orchard. New genomic technologies also offer the possibility of accelerating and increasing efficiencies and effectiveness of breeding programmes for new pear cultivars. Most pear fruit traits, such as texture, size, storage ability, resistance to scuffing, flavour and aroma are reported to be affected by numerous loci with small-to-moderate effects^3–6^.

A traditional MAS scheme ignores the contribution of all other genes, and thus could result in a lower response to selection, especially for polygenic traits. High-speed and reduced cost of genotyping technologies have facilitated the availability of large number of single nucleotide polymorphic (SNP) markers enabling researchers to study marker-trait associations across the whole genome. High-density genotyping platforms have also facilitated the implementation of genomic selection (GS). GS involves simultaneous estimation of genomewide SNPs effects to predict genomic breeding values^7^. The traditional MAS is best suited for monogenic or oligogenic traits whereas GS is ideal for traits controlled by many loci with small-to-moderate effects. Therefore, a two-stage selection strategy combining MAS and GS has the potential to accelerate breeding cycles and improve the efficiency of fruit breeding programmes^8^. There are reports of GWAS in germplasm populations of Japanese pear *P*. *pyrifolia*^6,9^ and *Pyrus* spp.^5^, but the evaluation of GS has only been attempted in Japanese pear^6^.

Despite sensory traits being primary selection criteria for developing new cultivars, there appears to be no report of GWA and GS for pear sensory traits – something that hinders the acceleration of breeding cycles. The main objectives of this study were to conduct GWA to find the candidate genomic regions for pear fruit traits including sensory eating quality traits, and evaluate the potential of GS using a hybrid population derived from crosses between Asian and European pears.

## Results

### Genetic parameters

The distribution of adjusted fruit phenotypes is shown in Supplementary Fig. S1. Estimates of narrow-sense heritability were low for sweetness (SWET, 0.16), but moderate–high (0.40–0.69) for all other traits (Table 1). Fruit weight (AVFW) was the most heritable trait at 0.69, followed by sourness (SOUR). The highest genetic correlation (0.81) was observed between sensory firmness (FIRM) and crispness (CRIS). Sourness (SOUR) was adversely correlated with sweetness (SWET: −0.31) and flavour intensity (FINT: −0.46), and SWET was favourably correlated (about 0.40) with juiciness (JUIC) and FINT. Fruit with high FIRM were relatively less susceptible to scuffing (SCUF) as there was a significant correlation (−0.24) between these traits. High russet (RUSS) fruit tended to display high SOUR and less JUIC. Estimated genetic correlation of AVFW with SOUR, FINT, SHAP and RUSS was found to be significant (Table 1).

**Table 1:** Genetic parameters of various pear fruit quality traits (firmness: FIRM; crispness: CRIS; juiciness: JUIC; sweetness: SWET; sourness: SOUR; flavour intensity: FINT; fruit scuffing: SCUF; shape: SHAP; russet: RUSS; fruit weight: AVFW). Diagonals are estimated narrow-sense heritability and off-diagonals are genetic correlations. Significant (*p* < 0.0001) correlations are marked with*.

	FIRM	CRIS	JUIC	SWET	SOUR	FINT	SCUF	SHAP	RUSS	AVFW
FIRM	**0**.**47**									
CRIS	0.81*	**0**.**40**								
JUIC	0.23*	0.39*	**0**.**58**							
SWET	0.09	0.21*	0.41*	**0**.**16**						
SOUR	−0.09	−0.11	−0.02	−0.31*	**0**.**62**					
FINT	0.17	0.10	0.26*	0.38*	−0.46*	**0**.**46**				
SCUF	−0.24*	−0.19*	−0.08	0.05	0.21*	−0.21*	**0**.**51**			
SHAP	−0.05	−0.05	−0.04	−0.07	0.06	−0.10	−0.12	**0**.**57**		
RUSS	−0.16	−0.15	−0.24*	−0.05	0.24*	−0.27*	−0.08	0.03	**0**.**59**	
AVFW	−0.10	0.04	0.05	0.12	0.30*	−0.32*	0.04	0.33*	0.28*	**0**.**69**

### Population structure and LD decay

**Figure 1:**
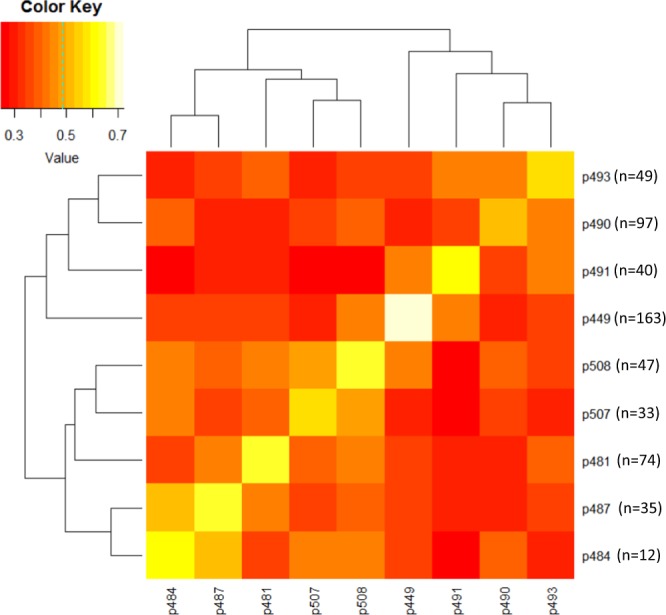
The average within- and between-family pairwise coefficient of relationships for various pear families. The number of offspring in each family (n) is also shown.

**Figure 2:**
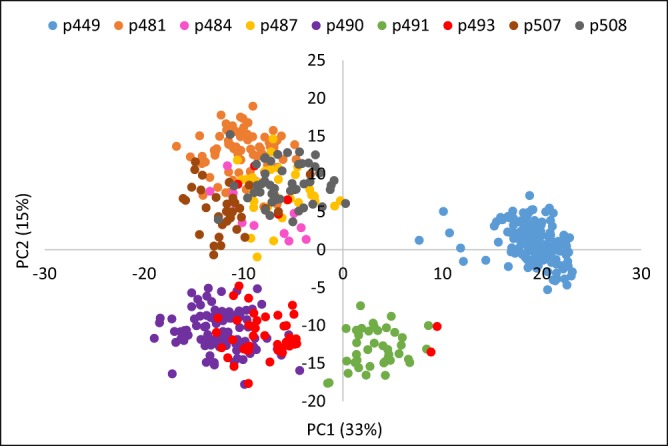
Principal component (PC) analysis plot of the first two components of 550 seedlings derived from their marker genotypes. Pedigree-based grouping (i.e. full-sib families) is also depicted in different colours.

**Figure 3:**
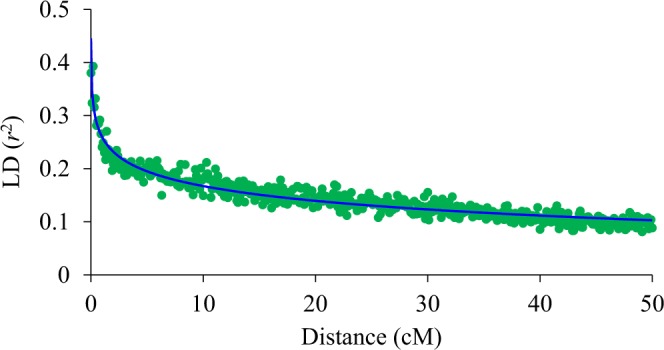
Average linkage disequilibrium (LD) measured as *r*^2^, for pairs of single nucleotide polymorphisms (SNPs) in increments of 0.1 cM, according to the distance between SNPs in the population of 550 seedlings.

Overall, a product-moment correlation of 0.69 was observed between pedigree-based and SNP-based estimates of pair-wise coefficient of relationships. The average SNP-based within-family pairwise relationship ranged from 0.50 (family p490) to 0.72 (p449), and the average relationships among seedlings from different families ranged from 0.30 and 0.52 (Fig. 1). Family p449 was derived from crossing two siblings, hence showed a relatively higher relationship coefficient. A plot of the first two principal components (PCs) of the SNP-based realized relationship matrix (***G***) grouped seedlings largely according to their familial relationships (Fig. 2). The first two PCs were used to account for population structure in GWA models. Some individuals did not cluster within their pedigree-assigned full-sib family groupings. For example, two individuals from family p493 clustered with p491, which suggested some pollen contamination or mislabelling. The pattern of LD (*r*^2^) decay in the genetically related population of 550 individuals showed a high degree of LD even at longer distances between markers. For example, the average *r*^2^ for SNPs separated by 0.5 cM, 1.0 cM, and 5.0 cM was 0.29, 0.26, and 0.19, respectively (Fig. 3).

### Genetic architecture

**Figure 4:**
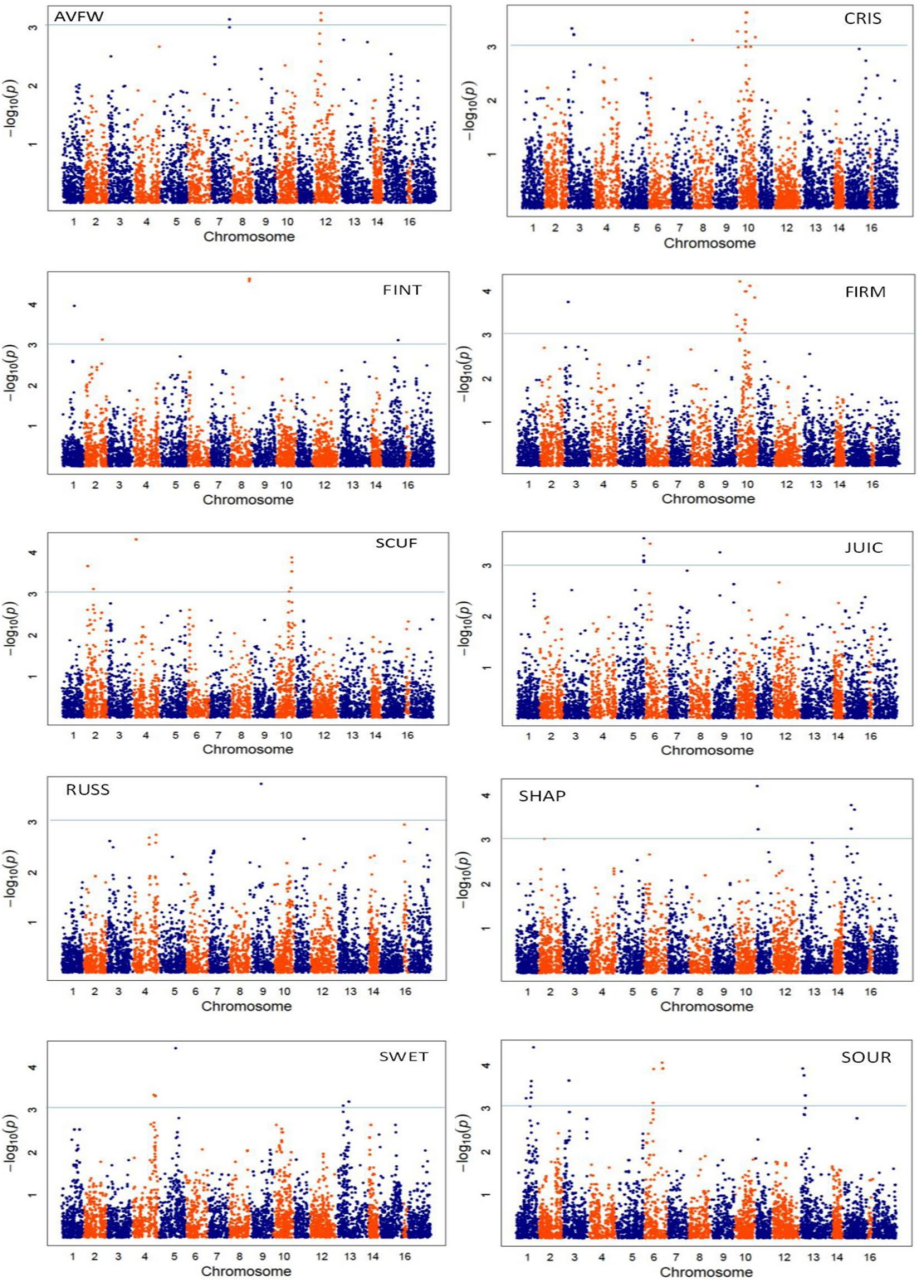
Manhattan plots of the −log_10_(*p*) values for various pear fruit traits (firmness: FIRM; crispness: CRIS; juiciness: JUIC; sweetness: SWET; sourness: SOUR; flavour intensity: FINT; fruit scuffing: SCUF; shape: SHAP; russet: RUSS; fruit weight: AVFW) from a genome-wide scan are plotted against position on each of 17 linkage groups. Blue horizontal line indicates the significance threshold *p* < 0.001.

**Figure 5:**
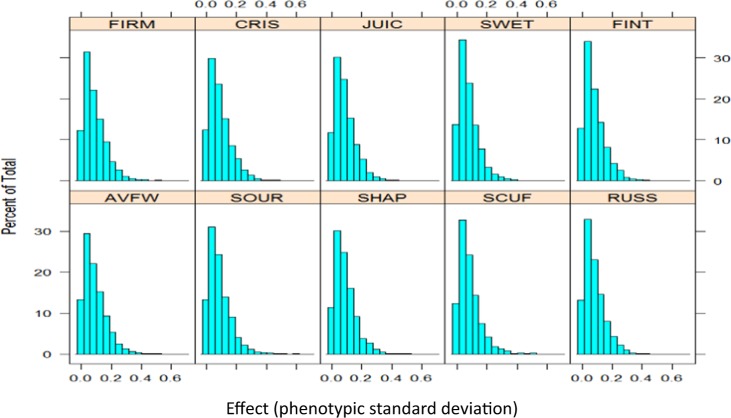
Histogram of allele substitution effects (in phenotypic standard deviation units) of SNPs for pear fruit quality traits (firmness: FIRM; crispness: CRIS; juiciness: JUIC; sweetness: SWET; sourness: SOUR; flavour intensity: FINT; fruit scuffing: SCUF; shape: SHAP; russet: RUSS; fruit weight: AVFW).

Using single-locus GWA, significant (*p* < 0.001) SNP-trait association signals for FIRM and CRIS were identified on LG3 and LG10; for SCUF on LG2, LG4 and LG10; for SHAP on LG11 and LG15; for SOUR on LG1, LG6 and LG13; for SWET on LG4, LG5 and LG13; and for FINT on LG1 and LG8 (Fig. 4). The majority of SNPs individually explained about 0.5% of phenotypic variance (Supplementary Fig. S2), while the maximum effect-size varied between 2% (for AVFW and RUSS) and 3.5% (SWET) (Table 2). The largest-effect SNP was common between FIRM and CRISP on LG10, while the SNPs with largest effect on JUIC and SWET were located on LG5 (Table 2). The distribution of observed ASEs (presented as phenotypic standard deviation (PSD)) for each trait were moderately leptokurtic, suggesting only few SNPs with moderate effect, and the highest ASE varied between 0.41 and 0.68 (Fig. 5). The Kolmogorov-Smirnov test showed that these observed distributions were significantly (*p* < 0.05) different from normal and exponential density functions, but fitted best to a gamma distribution (i.e. the majority of the SNPs having a small effect and a few a moderate effect). The estimated shape and rate parameters of gamma distribution were about 1.25 and 15.0, respectively, for all traits.

**Table 2:** Single nucleotide polymorphism (SNP) with the largest effect (phenotypic variance explained by the SNP; R^2^) on various pear fruit quality traits (firmness: FIRM; crispness: CRIS; juiciness: JUIC; sweetness: SWET; sourness: SOUR; flavour intensity: FINT; fruit scuffing: SCUF; shape: SHAP; russet: RUSS; fruit weight: AVFW). The allele substitution effect (ASE), measured in phenotypic standard deviation units, of the largest-effect SNP is also presented. Statistical significance of SNP effect is shown using −log_10_*p* values.

Trait	SNP	Linkage group	Position (cM)	Significance (−log_10_*p*)	ASE	R^2^ (%)
FIRM	S764_78213	10	95.60	4.00	0.52	3.0
CRISP	S764_78213	10	95.60	3.64	0.48	2.6
JUIC	S210_30053	5	298.97	3.54	0.41	2.2
SWET	S182_250115	5	189.89	4.45	0.33	3.5
SOUR	S465_110990	1	187.40	4.42	0.44	3.0
FINT	S203_236962	8	214.20	4.63	0.41	3.3
SCUF	S29076_1553	10	184.92	3.88	0.44	2.4
SHAP	S4855_850	11	16.65	4.21	0.65	3.0
RUSS	S150_272459	9	123.17	3.77	0.48	2.0
AVFW	S340_202551	12	79.42	3.25	0.53	2.0

The number of significant (*p* < 0.001) SNPs identified using multi-locus methods MLMM and MRMLM were 79 and 77, respectively, compared to 67 identified from single-locus GWA (Supplementary Table S1). Majority of the significant SNPs identified using the two multi-locus methods were on the same genomic locations as those from single-locus method (GAPIT) for all traits (Supplementary Figs S3 and S4, Supplementary Table S1). MLMM and MRMLM identified SNPs significantly associated with RUSS on LG 4, 16 and 17, which were insignificant in single-locus GWA (Supplementary Table S1).

### Genomic prediction accuracies

**Figure 6:**
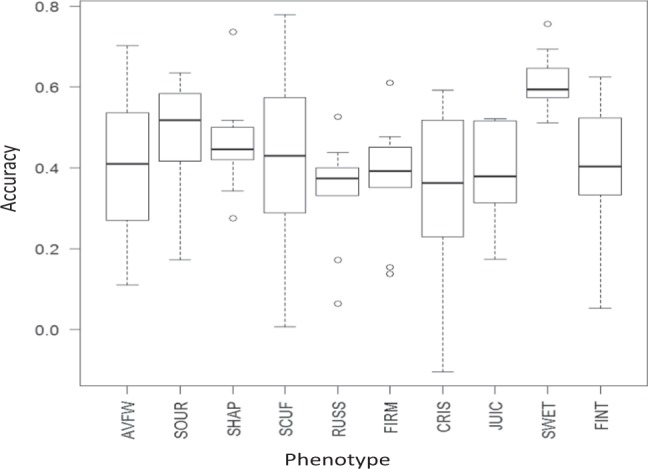
Accuracy of genomic selection for pear fruit quality traits (firmness: FIRM; crispness: CRIS; juiciness: JUIC; sweetness: SWET; sourness: SOUR; flavour intensity: FINT; fruit scuffing: SCUF; shape: SHAP; russet: RUSS; fruit weight: AVFW).

We applied a cross-validation scheme by using each full-sib family in turn as a validation population (VP), resulting in a nine-fold cross validation. The results are displayed using a boxplot graph (Fig. 6). The average (across nine families) accuracy varied from 0.32 (CRIS) to 0.62 (SWET), and the range of predicted accuracy was lowest (0.24) for SWET and highest (0.77) for SCUF. The higher prediction accuracy for SWET was partly due to low genetic and phenotypic variability (Table 1, Supplementary Fig. 1). Across all 10 traits, the prediction accuracy was lowest (0.32) for family p449 and the highest (0.51) for family p487.

## Discussion

Understanding of the heritability (*h*^2^) of a selection trait is critical in designing molecular breeding strategies such as MAS and GS^7,8^. Using genomic relationship matrix (GRM) in mixed-model equations would provide improved estimation of *h*^2^ compared to the pedigree-based relationships^10^. For a quantitative trait controlled by many genes, the proportion of *h*^2^ explained by a SNP would indicate its worthiness for use in traditional MAS. Most traits in this study were under moderate-to-high genetic control, with GRM-based estimates of *h*^2^ being comparable with earlier published pedigree-based estimates. For example, *h*^2^ of fruit weight (0.68) and acid taste (0.62) were almost identical to those reported by Minamikawa *et al*.^6^. The, *h*^2^ for fruit firmness in interspecific populations has been reported to be around 0.60–0.70^11,12^, slightly higher than that found in this study (0.47). Heritability of FINT in interspecific populations was reported to be 0.54^13^, which is similar to that observed in this study (0.46). However, *h*^2^ of fruit sweetness as reported here (0.16) was half of that observed by Abe *et al*.^14^ in hybrid seedlings. In our study, the average (over traits) GRM-based *h*^2^ was 0.50, which was slightly lower than the pedigree-based estimate (0.60). In general, GRM-based *h*^2^ were shown to be in the similar range to pedigree-based estimates in various species^15–17^, which is consistent with results from this study.

LD is a measure of associations between SNP alleles and the alleles at QTL^18^. In practice, LD between pairs of SNP markers are obtained because the genomic coordinates of QTLs are generally unknown. The extent of LD obtained in the hybrid seedling populations in this study is higher than that in a population of Asian and European pear germplasm accessions^5^. For markers separated by 10 cM, the *r*^2^ (0.17) in our study is almost identical to that reported (0.18) by Minamikawa *et al*.^6^ in full-sib progenies of Japanese pear. The average *r*^2^ between the adjacent SNPs was 0.47, which is higher than that reported for pear (0.33)^6^ and apple (0.32)^17^.

The higher magnitude of short-range and long-range LD in our study could be a result of the genetic structure of the seedling population. Clustering patterns of families (Fig. 2) reflected sharing of parents between the various families. Strong relatedness between different families, and a bottleneck in the breeding history of parents of these families, could be among the factors underpinning the high observed LD^9,18^. High LD between the adjacent SNPs also plays a key role in improved accuracy of GS^19^.

Peak association signals for various traits were located close to genomic regions that have been previously identified. For example, putative QTLs identified for SCUF on LG2, 4 and 10 are in agreement with earlier reports^5,20^. Similar to our study, there are reports of QTLs for pear fruit shape index on LG2 and LG11^3,21^. Cao *et al*.^22^ reported a large effect QTL for apple fruit shape index on LG11, suggesting an orthologous region between apple and pear genomes. A QTL for AVFW was mapped on LG7^21^, which agrees with our results, but some studies^3,4^ mapped QTLs for AVFW on different linkage groups – suggesting this trait has a complex polygenic nature. As might have been expected given the high genetic correlation between FIRM and CRIS, these traits shared the largest-effect SNPs located on LG10 and LG3. Genomic locations on LG10 and LG3 have previously been shown to be associated with fruit firmness in apple^8,17,23^. QTLs influencing apple and pear fruit traits (e.g. fruit softening; harvest maturity) have been mapped on the same LGs of apple and pear genomes^6,24^ – further evidence for high synteny between pear and apple genomes^25,26^.

The same SNP marker was found to be associated with SWET and JUIC on LG5. A significant genetic correlation (0.41) between these traits (Table 1) would suggest some genes with pleiotropic effects. There are no reports on QTLs for pear SWET, but QTLs for soluble solids concentration (SSC) have been investigated as SSC is the best objective predictor of sensory sweetness^27^. In agreement with results of this study, QTLs for SSC have been mapped on LG4^4^ and LG5^3,21^. A high correlation was reported between malic acid content and SOUR taste in apple fruit^27^, hence common QTLs for these fruit traits would be expected. Minamikawa *et al*.^6^ mapped a large-effect QTL for acid content in pear fruit on LG6, which supports our GWA results. Unlike our study, there are no QTLs reported for SOUR taste on LG1 and LG13 – suggesting a possibility of population-specific QTL^28^. Similar to apple FINT QTL^29^, significant marker-trait associations were observed for pear FINT on LG1 and LG8. SNPs within the apple genes *MdCXE4* (LG1) and *MdMYB44* (LG8) were shown to be associated with apple FINT and/or fruit acid content^29,30^. Further work is needed to identify and test the functionality of putative candidate genes underpinning marker-trait associations reported in this study, and evaluate their synteny with apple genes.

Multi-locus GWA could be more powerful than single-locus GWA because they account for LD between SNPs, and a small-effect locus may be more apparent when other large-effect loci are already fitted in the model^31,32^. Similar to a previous study^6^ on pear GWA, a higher number of significant SNPs were detected in the multi-locus GWA compared to the single-locus analysis. However, the majority (nearly 90%) of the significant SNPs, especially the large-effect SNPs, were common between the two approaches in our study (Supplementary Table S1). As some small-effect SNPs (e.g. for RUSS) were not detected in the single-locus model, multi-locus GWA could be more powerful in such cases. However, it’s important to note that the threshold (*p* < 0.001) used to identify significant SNPs in our study is low and the false-discovery rate adjusted *p*-values were >0.05 for most traits. These results emphasise that an independent validation of the significant SNPs would be required irrespective of the GWA method used.

Understanding the genetic architecture of pear fruit phenotypes is facilitated by having information regarding the distribution of QTL effects. The variance explained by a SNP is mainly a function of the size of the QTL associated with the significant marker and LD between the marker and QTL^7^. Consequently, the distribution of estimated SNP effects should resemble the distribution of the underlying QTL effects. In this study, the maximum variance explained by a SNP for any trait was very small (<4%) and there were very few SNPs with ASE higher than 0.50 PSD, so gamma distributions best fitted to the observed SNP effects for all fruit traits. These results indicated that many QTLs of small effect and only a few moderate effect QTLs control the fruit traits investigated in this study. Hayes *et al*.^33^ also reported a gamma distribution of QTL effect sizes in dairy and pig breeding programmes; this is supported by similar observations in other species^34,35^. Our results suggest that the response from MAS in most pear fruit phenotypes would be small – hence GS could be a better selection tool. MAS would still be useful for traits controlled by major genes, such as pear red skin colour^36^.

The fundamental difference between MAS and GS is that the former only utilizes the SNPs that are significant in a GWAS, whereas the latter uses high-density genome-wide SNPs so that all QTLs are expected to be in LD with one or more SNPs. In the case of GS, potentially all the genetic variance for a trait can be tracked because the marker effect does not need to exceed a pre-determined significance threshold to be used to predict breeding value^7^. In fruit breeding programmes, the traditional MAS for major gene traits (e.g. disease resistance, skin/flesh colour) is followed by orchard testing for polygenic traits – hence the time required for developing new cultivars is not shortened to any great extent. A two-step approach, i.e. MAS for monogenic traits followed by GS for polygenic traits, obviates the need for Stage-1 seedling testing, hence fast-forwarding the development of new cultivars^8^. This strategy has been evaluated in the New Zealand-based PFR apple breeding programme, and it could be adapted to pear breeding programmes as well.

Various studies on apple have shown that the correlation between sensory and instrumental measures of firmness, acidity, and sweetness were about 0.75, 0.80 and 0.50, respectively^27,37^, suggesting that genomic prediction accuracies of sensory and instrumental measures could be similar. Minamikawa *et al*.^6^ used instrumental measures of some of the sensory traits used in our study. The average prediction accuracy of traits (fruit weight, firmness, sweetness, and sourness), that were common with Minamikawa *et al*.^6^, was almost identical (cf. 0.46). For the across-family validation scheme implemented in this study, the genetic relationships between the training and validation families and population-level LD are among the key drivers of genomic prediction accuracy. Genomic predictions were reported to be most accurate when models were trained with some individuals from the validation families because of the close relation between training and validation sets^8,38^. Prediction accuracies of pear fruit phenotypes could be improved further by increasing the training population size, and also by combining parental and breeding populations^6^.

When the training and validation samples are observed independently over different sites/environments, prediction accuracies can be lower depending on the magnitude of genotype-by-site interaction^39^. Training data would need to be obtained from different sites and years in order to develop robust genomic prediction models for pear fruit traits. A common approach for the evaluation of GS in fruit breeding programmes is to cross-validate using validation samples from the same generation^6,8,39,40^. The accuracy of predicting phenotypes of successive generations would be lower than within-generation accuracy due to marker-QTL LD decay^19^. Meuwissen *et al*.^41^ suggested that Bayesian GS models could outperform GBLUP because they capture marker-trait LD that persists in the successive breeding cycles. However, various studies^42,43^ based on empirical data suggested very little or no advantage of Bayesian GS models for most traits, so GBLUP method is widely adopted for GS in commercial breeding programmes^44,45^.

Similar to animal and plant species^7,46,47^, fruit breeding programmes need to develop unique multi-generation genotype-phenotype datasets to evaluate persistence of accuracy of genomic predictions over several generations under different environmental conditions. Based on the accuracy of GS in our study, we conclude that it shows strong potential to accelerate the pear breeding cycle by making selections prior to extensive fruit-quality phenotyping. Thus, a GWAS-GS combination could be an effective tool for increasing the efficiency of pear breeding programmes.

## Method

### Plant material

An interspecific pear breeding programme at Plant & Food Research Limited (PFR) New Zealand was initiated in 1986 using commercial cultivars of European, Chinese and Japanese pear as parents. Second-generation populations were created in 1996 using the best selections produced from the first-generation hybrid families as parents^36^. A subset of the third-generation families created during 2007–08 using best seedlings from the second-generation were used for this study. A total of 12 second-generation selections were used as parents to create nine families and details of mating design and relatedness among the 12 parents were reported earlier^36^.

As described earlier by Kumar *et al*.^36^, a random subset of seedlings that reached a minimum height after growing in a field nursery for 1 year were propagated and planted in PFR’s orchard in Motueka during 2011. Fruit were harvested during the fruiting season in 2015 and 2016 and a random sample of six fruit from each seedling was stored for 28 days at 3 °C, then a further 1 day at 20 °C before evaluation^36^. Sensory traits including firmness (FIRM), crispness (CRISP), juiciness (JUIC), sweetness (SWET), sourness (SOUR) and flavour intensity (FINT) were evaluated on a scale from 0 (=lowest) to 9 (=highest) by two trained assessors, and one overall score for each trait was given to each seedling. Russet (RUSS) was scored visually on a scale from 0 (=lowest) to 9 (=highest). Scuffing (SCUF) was rated on a 0–9 scale (0 = no darkening; 9 = solid brown or black colouration) following the method described by Brewer *et al*.^48^. Fruit shape index (SHAP) was measured using a two dimensional shape chart^5^ and fruit weight (AVFW) was measured as the average weight of the six fruits.

### DNA extraction, variants discovery, and linkage map construction

Protocols for DNA extraction, genotyping-by-sequencing (GBS) library preparation, and variant calling were reported earlier as the SNP data used in this study are those used for fine-mapping of pear red skin colour gene in the same nine families^36^. Resulting SNPs were used to construct a consensus linkage map as reported earlier by Kumar *et al*.^36^. Briefly, a total of 16 paternal and maternal maps were constructed using *Joinmap* v4.1 software, and then common SNPs shared by at least three maps were selected as a bridge to merge the maps using R package *MergeMap*. Finally, 7,509 high quality SNPs were mapped and used for GWA and GS in this study.

### Genomic BLUP model

Phenotypes adjusted for fixed effects (e.g. year and assessor effect) were used for estimation of variance components and best linear unbiased prediction (BLUP) of additive effects using the following model^17^:

1 $${\boldsymbol{y}}=\mu {{\bf{1}}}_{{\bf{n}}}+{\boldsymbol{Za}}+{\boldsymbol{e}}$$

where ***y*** is a vector of adjusted phenotypes; μ is an intercept, **1**_**n**_ is a vector of 1 s; ***Z*** is the known design matrix relating to ***a***, the unknown vector of random additive genetic effects with ***a*** ~ *N*(0, ***G*** ${\sigma }_{a}^{2}$). The scalar ${\sigma }_{a}^{2}$ is the additive variance and ***e*** is a vector of independent random deviates with variance ${\sigma }_{e}^{2}$. A realized (or genomic) relationship matrix (***G***)^49^ was obtained using all available SNPs, and Eq. (1) was implemented in software ASReml v3.0^50^. Estimates of variance components derived from Eq. (1) were used for calculating narrow-sense heritability (*h*^2^) as the ratio of additive (${\sigma }_{a}^{2}$) to phenotypic variance ($={\sigma }_{a}^{2}+{\sigma }_{e}^{2}$). Product-moment correlations between breeding values were used as estimates of genetic correlation among various traits.

### Model validation

The dataset in this study was composed of nine families, so each family in-turn was used as a validation population (VP) and the remning eight families were used as a training population (TP). GBLUP of seedlings in a VP were predicted by fitting Eq. (1) where their phenotypes were considered as missing values. Prediction accuracy was obtained as the correlation between observed and predicted BLUP of individuals in the VP. This process was repeated so that all nine families were predicted. The mean (averaged over nine validation sets) accuracy and its standard error were presented for each trait.

### Estimation of linkage disequilibrium (LD) and genetic architecture

Estimates of LD (*r*^2^), derived from allele frequencies at a pair of loci, were calculated between SNPs located on the same linkage group^36^. The *r*^2^ estimates were corrected for population structure and cryptic relatedness, and then the average across LGs were plotted against pairwise genetic distance using R package LdcorSV^51^. The LD decay curve was fitted using a standard logarithmic function. Genetic architecture, in terms of allele substitution effects (ASE) of genome-wide SNPs, of each trait was investigated using the single-locus unified mixed linear model (MLM) approach that accounts for family structure and cryptic relationships as implemented in R package GAPIT^52^. The following MLM was implemented for single-locus GWA^52^:

2 $${\boldsymbol{y}}=X{\boldsymbol{\beta }}+{\boldsymbol{Za}}+{\boldsymbol{\varepsilon }}$$

where ***y*** is a vector of adjusted phenotypes; **β** is an unknown vector containing estimates of fixed effects (overall mean, ASE of the SNP, and population structure); ***X*** and **Z** are the known design matrices relating to **β** and ***a*** (the unknown vector of random additive genetic effects with variance ***G*** ${\sigma }_{a}^{2}$), respectively. The scalar ${\sigma }_{a}^{2}$ is the additive variance, ***G*** is the realised or genomic relationship matrix (GRM)^49^, and ***ε*** is a vector of independent random deviates with variance ${\sigma }_{\varepsilon }^{2}$. The estimated ASEs of all SNPs were expressed in phenotypic standard deviation units and the observed distribution of SNP effects was compared with theoretical density functions (Normal, Gamma, Exponential) using Kolmogorov-Smirnov test implemented in R package ‘fitdistrplus’^53^. The proportion of phenotypic variance explained by each SNP was also calculated using R package GAPIT.

Multi-locus GWA methods could potentially be more powerful than single-locus GWA especially for complex traits. Two multi-locus methods were evaluated in this study; first, a multi-locus mixed model (MLMM^31^) which accounts for population structure and cryptic relatedness and uses a stepwise regression with forward inclusion and backward elimination of SNPs as fixed cofactors; second, a multi-locus random-SNP-effect mixed linear model (MRMLM^32^) which fits all SNPs simultaneously as random effects and also accounts for population structure and cryptic relatedness (https://cran.r-project.org/web/packages/mrMLM.GUI/index.html).

## Supplementary information

Supplementary Figures and Tables
