# Supplementary material for: Marker-trait associations and genomic predictions of interspecific pear (Pyrus) fruit characteristics
Source: Sci Rep. 2019 Jun 21;9:9072. doi: 10.1038/s41598-019-45618-w (PMC6588632; doi:10.1038/s41598-019-45618-w)
Supplement: Supplementary file 1 — Supplementary Figures and Tables [file 41598_2019_45618_MOESM1_ESM.pdf]

Supplementary Information

Marker-trait associations and genomic predictions of interspecific pear (*Pyrus*) fruit characteristics

Satish Kumar[1*], Chris Kirk[2], Cecilia Hong Deng[3], Angela Shirtliff[5], Claudia Wiedow[2], Mengfan Qin[4], Jun Wu[4], Lester Brewer[5]

[1]The New Zealand Institute for Plant and Food Research Limited, Hawke's Bay Research Centre, Havelock North, New Zealand.
[2]The New Zealand Institute for Plant and Food Research Limited, Palmerston North Research Centre, Palmerston North, New Zealand.
[3]The New Zealand Institute for Plant and Food Research Limited, Mount Albert Research Centre, Auckland, New Zealand.
[4]Centre of Pear Engineering Technology Research, Nanjing Agricultural University, Nanjing 210095, China
[5]The New Zealand Institute for Plant and Food Research Limited, Motueka Research Centre, Motueka, New Zealand.

[*]Corresponding author:
Satish Kumar: satish.kumar@plantandfood.co.nz

Supplementary Figures S1- S4

Supplementary Table S1

Supplementary Fig. S1. Distribution of various pear fruit quality traits (firmness: FIRM; crispness: CRIS; juiciness: JUIC; sweetness: SWET; sourness: SOUR; flavour intensity: FINT; fruit scuffing: SCUF; shape: SHAP; russet: RUSS; fruit weight: AVFW).

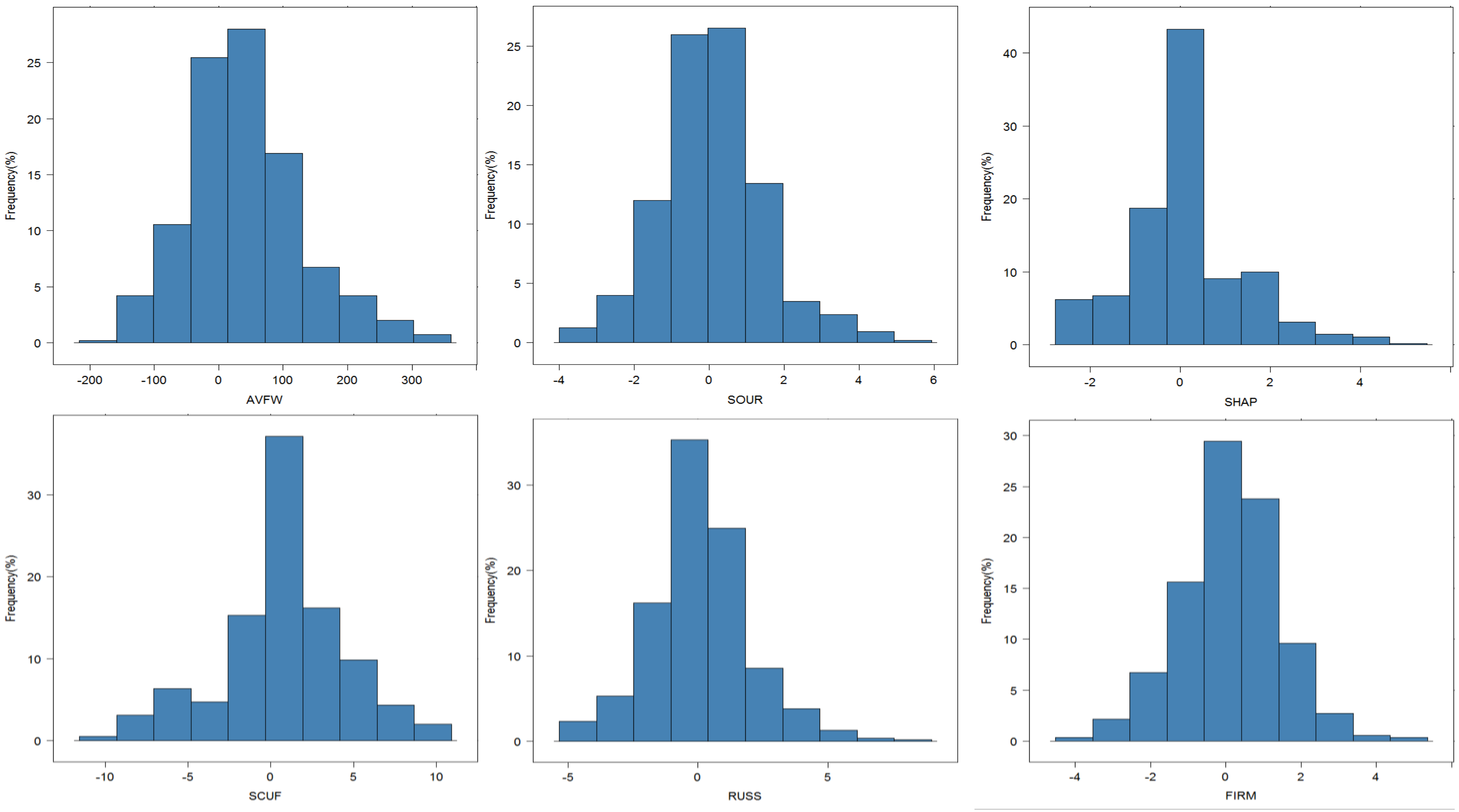

Supplementary Fig. S1 (continued)

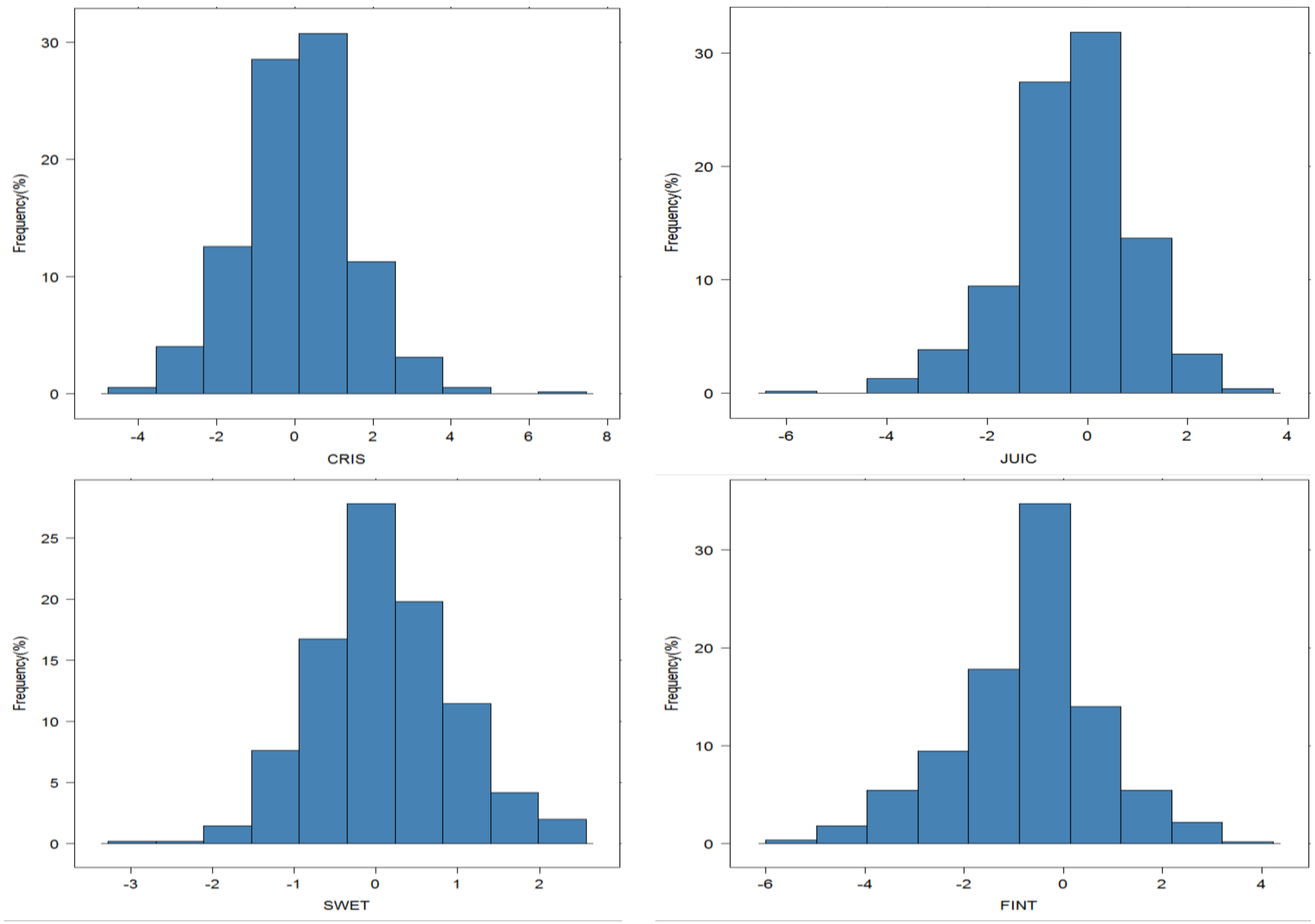

Supplementary Fig. S2. Effect size (proportion of phenotypic variance explained) of genome-wide SNPs for various pear fruit quality phenotypes (firmness: FIRM; crispness: CRIS; juiciness: JUIC; sweetness: SWET; sourness: SOUR; flavour intensity: FINT; fruit scuffing: SCUF; shape: SHAP; russet: RUSS; fruit weight: AVFW).

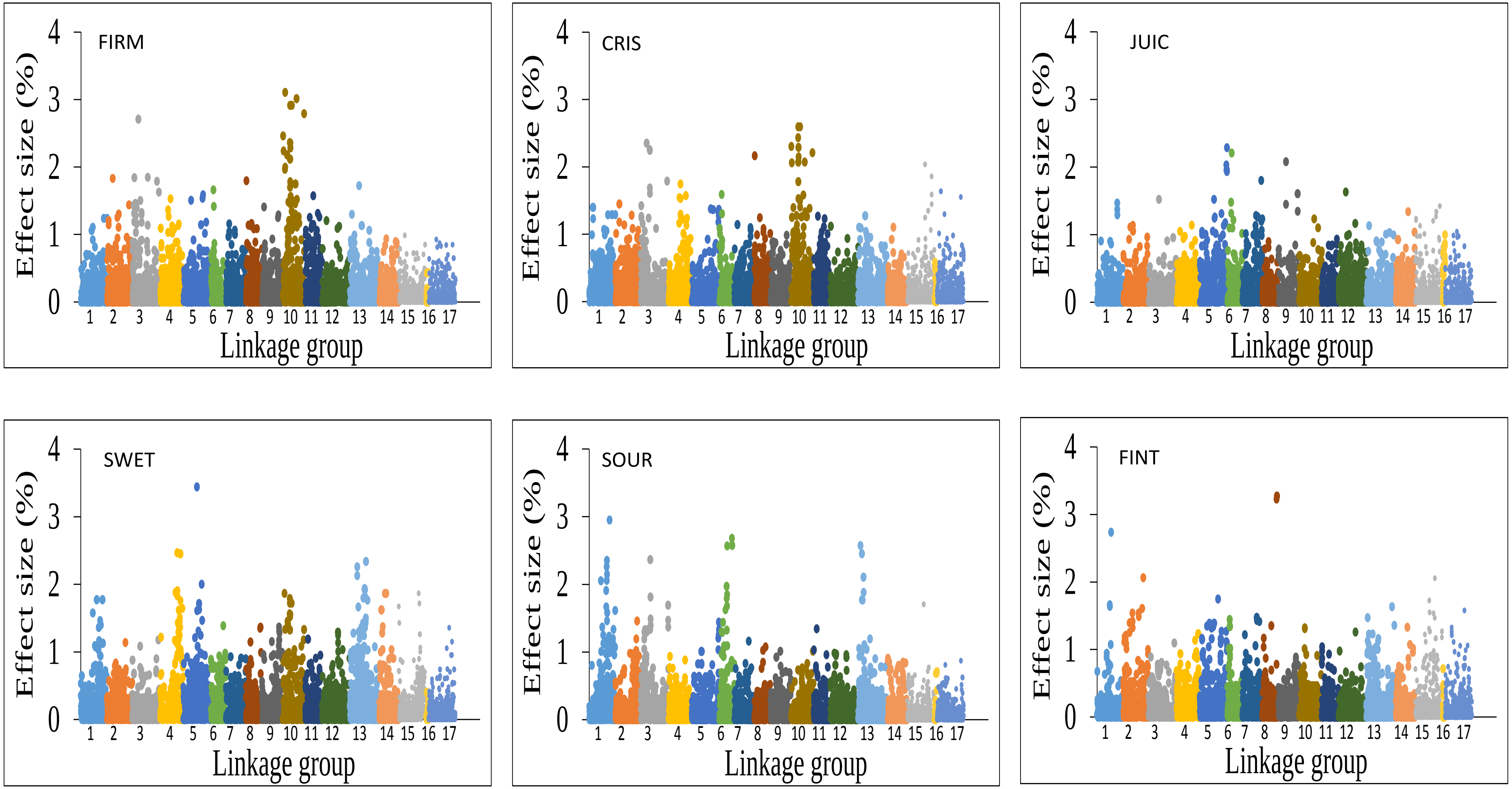

Supplementary Fig. S2 (continued)

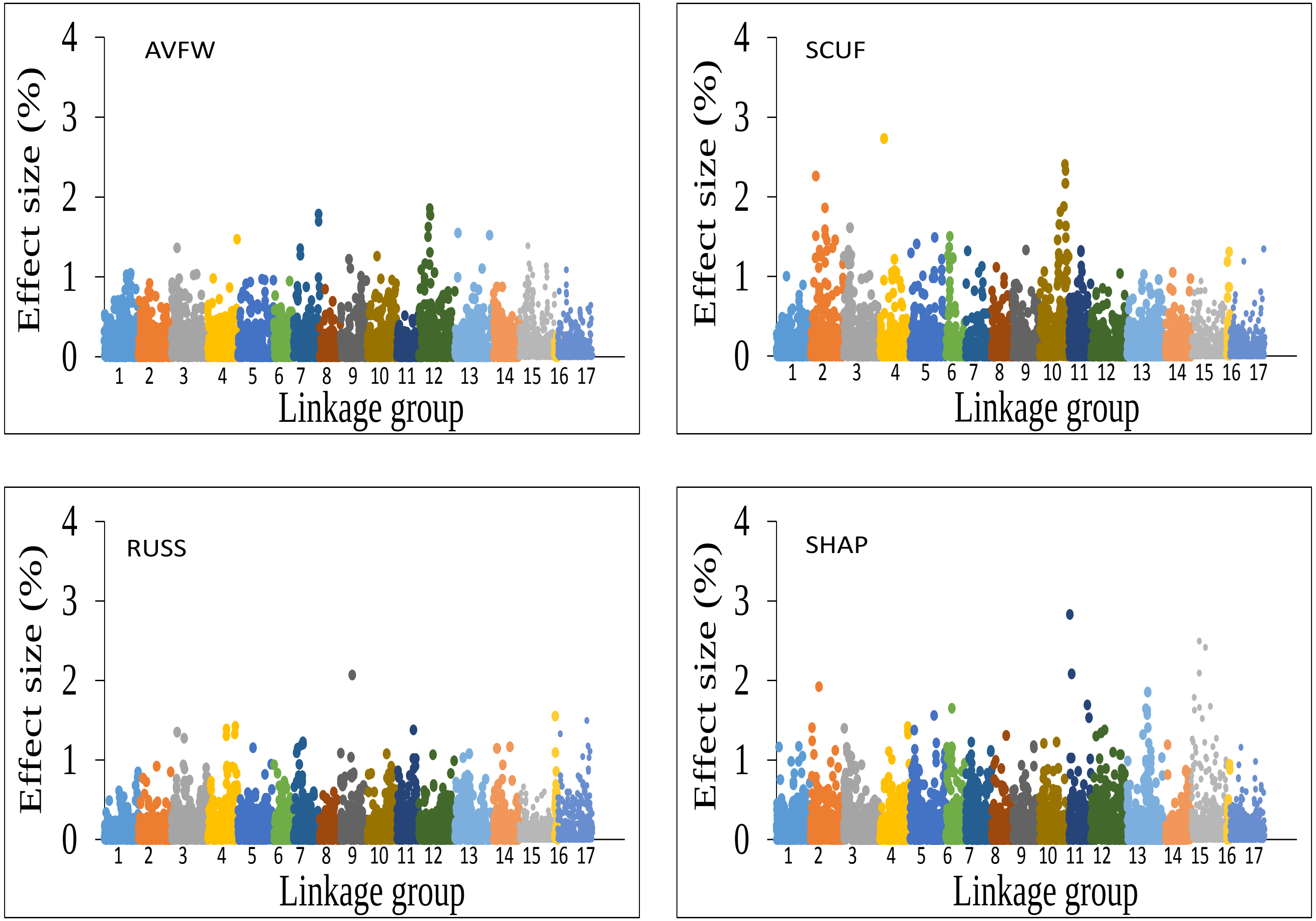

Supplementary Fig. S3. Manhattan plots of the –$\log_{10}(p)$ values (y-axis) for various pear fruit traits (firmness: FIRM; crispness: CRIS; juiciness: JUIC; sweetness: SWET; sourness: SOUR; flavour intensity: FINT; fruit scuffing: SCUF; shape: SHAP; russet: RUSS; fruit weight: AVFW) from a genome-wide scan (using multiple SNPs as fixed co-factors; **MLMM**) are plotted against position on each of 17 linkage groups (x-axis). Blue horizontal line indicates the significance threshold $p < 0.001$.

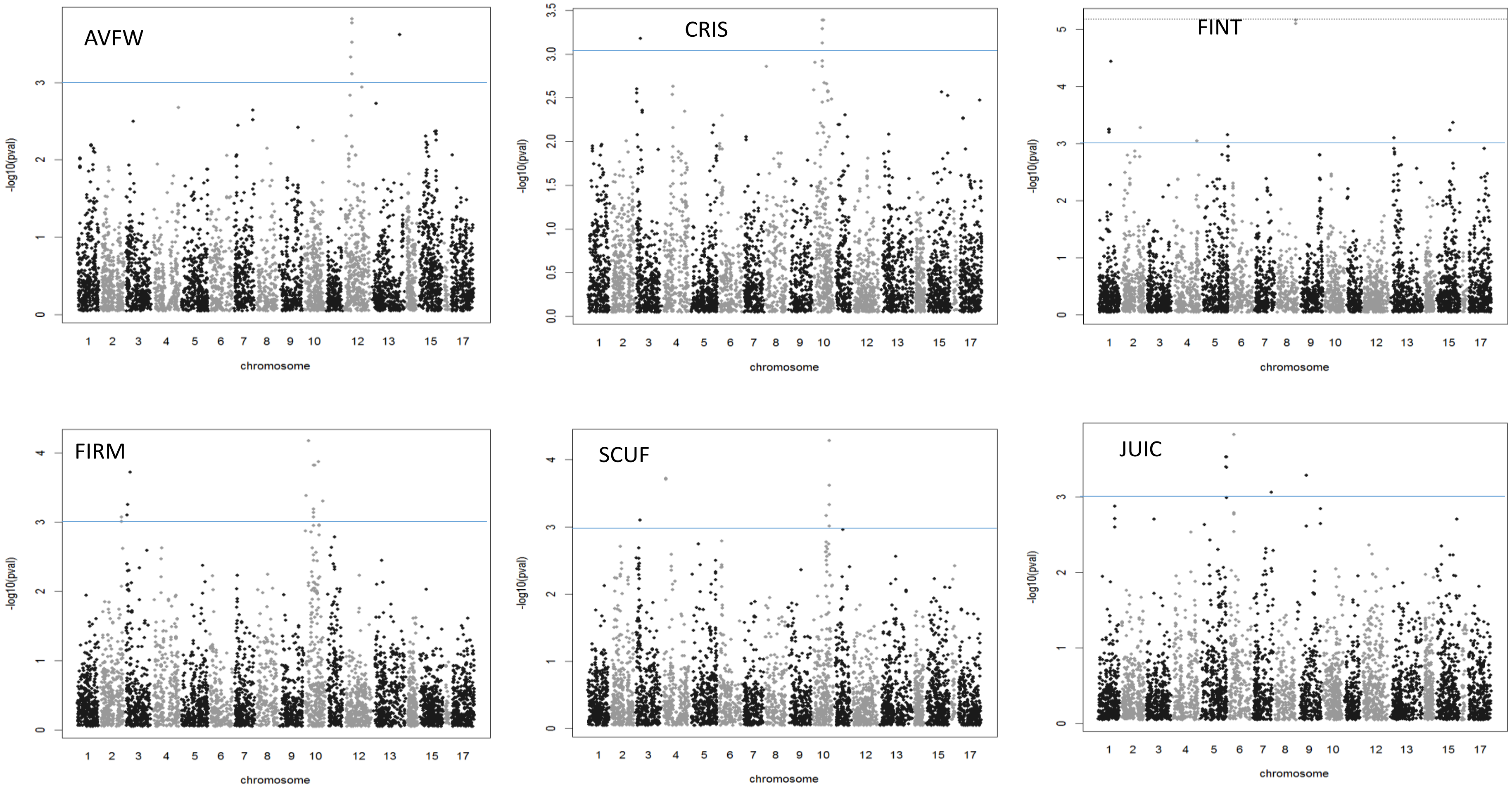

Supplementary Fig. S3 (continued)

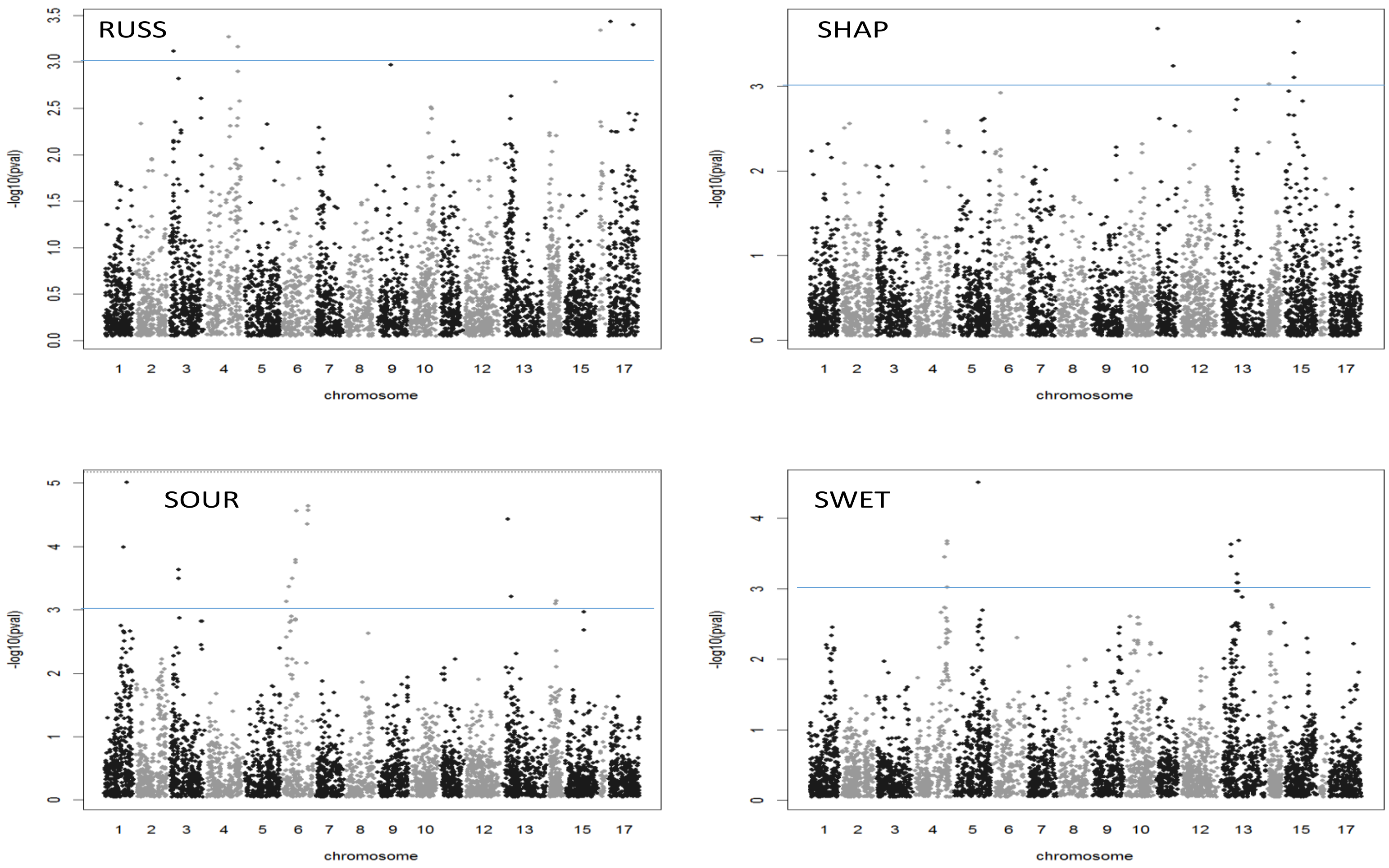

Supplementary Fig. S4. Manhattan plots of the $-\log_{10}(p)$ values (y-axis) for various pear fruit traits (firmness: FIRM; crispness: CRIS; juiciness: JUIC; sweetness: SWET; sourness: SOUR; flavour intensity: FINT; fruit scuffing: SCUF; shape: SHAP; russet: RUSS; fruit weight: AVFW) from a genome-wide scan (using multi-locus random model; **MRMLM**) are plotted against position on each of 17 linkage groups (x-axis). Blue horizontal line indicates the significance threshold p <0.001.

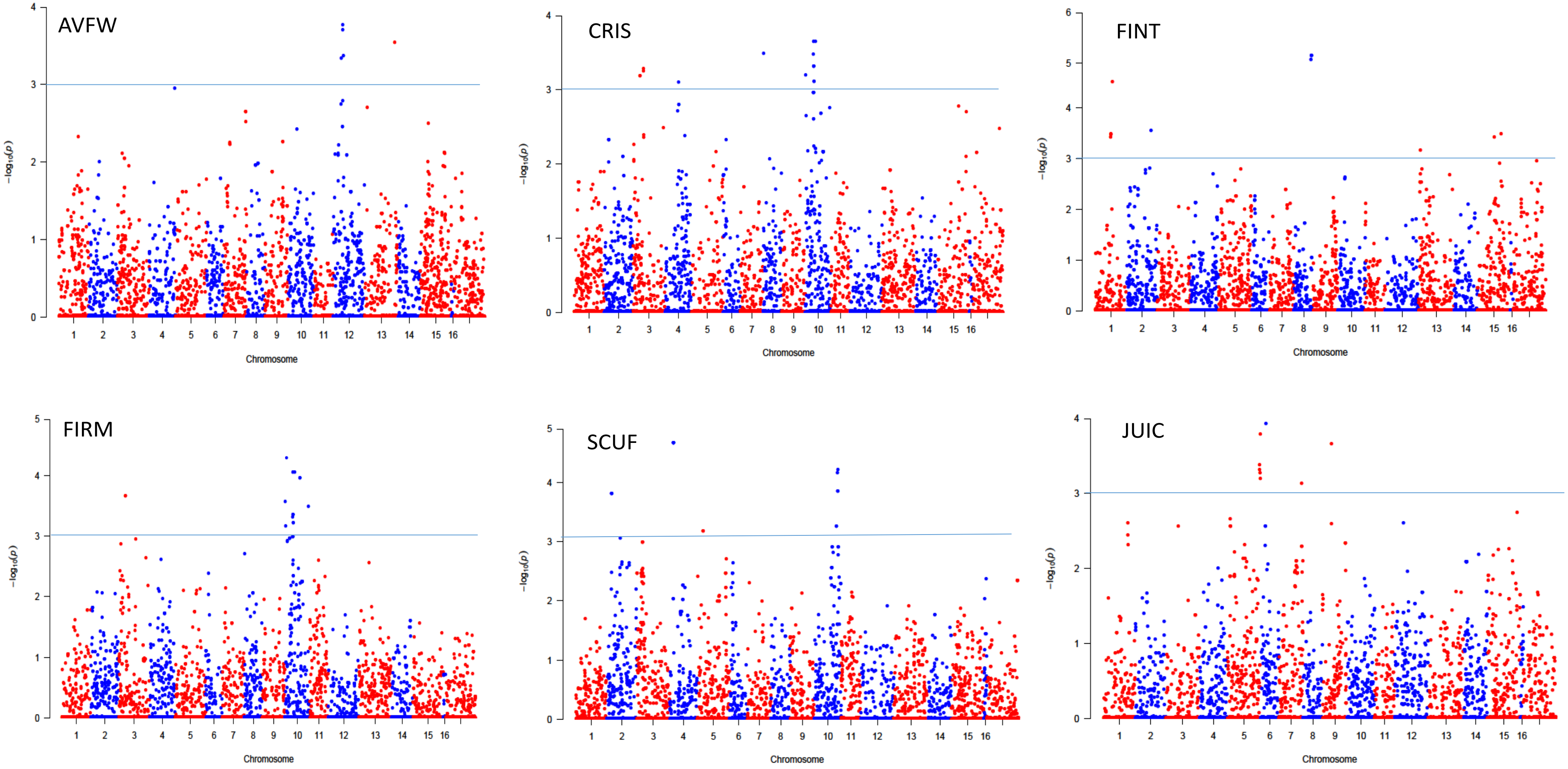

Supplementary Fig. S4 (continued)

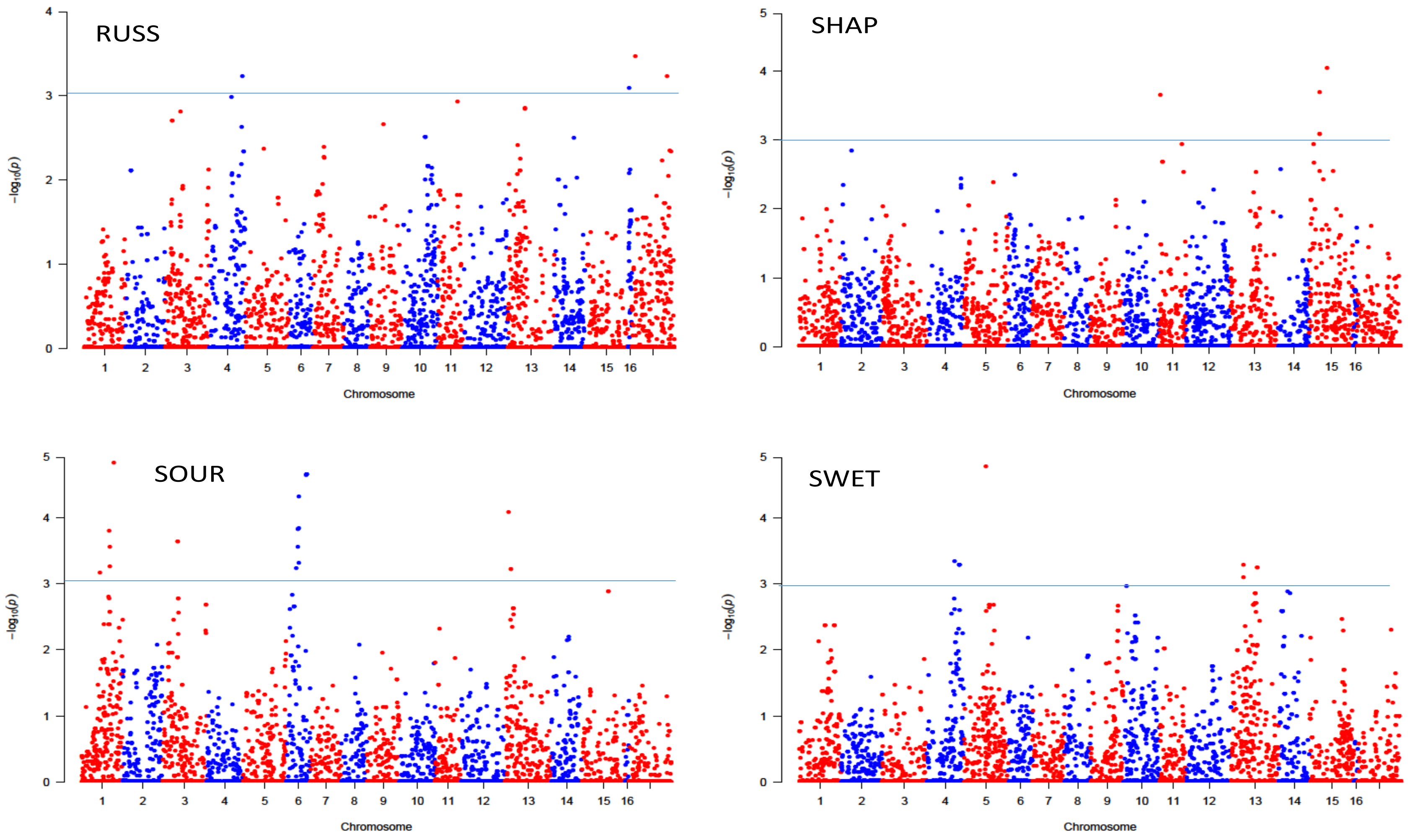

Supplementary Table S1. Significant SNPs detected by single-locus (GAPIT: Lipka et al. 2012) and multi-locus (MLMM: Segura et al. 2012; MRMLM: Wang et al. 2016) GWAS in interspecific pear population.

Trait	Method	SNP_ID	Linkage group	Position (cM)	-Log$_{10}$p	Minor allele frequency
AVFW	GAPIT	S90377_61	7	210.9	3.11	0.18
	MLMM	S340_157440	12	80.04	3.14	0.14
	MLMM, MRMLM	S340_157444	12	65.41	3.32, 3.34	0.34
		S281_132812	13	305.22	3.62, 3.55	0.35
	GAPIT, MLMM, MRMLM	S340_202551	12	79.42	3.25, 3.82, 3.77	0.11
		S340_157122	12	79.74	3.13, 3.76, 3.70	0.11
		S77_264162	12	81.43	3.12, 3.52, 3.37	0.33
CRIS	GAPIT	S587_89961	10	201.31	3.18	0.49
	MRMLM	S488_142340	4	111.87	3.11	0.07
	GAPIT, MRMLM	S136_172548	3	67.59	3.23, 3.25	0.13
		S527_123054	8	9.32	3.12, 3.49	0.2
		S278_9677	10	2.55	3.29, 3.20	0.24
		S295_219431	10	97.22	3.10, 3.11	0.19
	GAPIT, MLMM, MRMLM	S764_78213	10	95.6	3.64, 3.39, 3.65	0.2
		S961_23735	10	94.83	3.45, 3.29, 3.49	0.2
		S295_219507	10	96.55	3.27, 3.12, 3.32	0.19
		S1220_32314	3	46.33	3.35, 3.18, 3.19	0.41
FINT	MLMM	S330_62686	4	251.68	3.04	0.1
		S210_30068	5	288.39	3.15	0.18
	MLMM, MRMLM	S179_136748	1	117.22	3.25, 3.48	0.16
		S89_228187	15	143.03	3.23, 3.43	0.3
		S191_90827	13	33.61	3.10, 3.17	0.09
	GAPIT, MLMM, MRMLM	S203_236962	8	214.2	4.63, 5.16, 5.15	0.37
		S203_236958	8	211.56	4.59, 5.10, 5.08	0.36
		S94571_573	1	133.11	3.98, 4.44, 4.65	0.25
		S301_152577	2	205.11	3.13, 3.28, 3.55	0.13
		S118_262042	15	180.89	3.12, 3.37, 3.49	0.11
FIRM	MLMM	S7274_227	2	234.86	3.07	0.11
		S23_486843	3	16.39	3.1	0.13
		S744_151224	3	18.47	3.26	0.17
	MLMM, MRMLM	S278_9677	10	2.55	3.46, 3.57	0.24
	GAPIT, MLMM, MRMLM	S1220_32314	3	46.33	3.76, 3.72, 3.66	0.41
		S57756_421	10	11.18	3.20, 3.38, 3.17	0.44
		S41732_123	10	38.74	4.22, 4.18, 4.36	0.17
		S961_23735	10	94.83	3.34, 3.13, 3.32	0.2
		S764_78213	10	95.6	4.00, 3.82, 4.05	0.2
		S295_219507	10	96.55	3,34, 3.19, 3.35	0.19
		S295_219431	10	97.22	3,25, 3.07, 3.22	0.19
		S18_341916	10	147.99	4.12, 3.87, 3.96	0.16
		S587_89961	10	201.31	3.85, 3.30, 3.49	0.49

Supplementary Table S1 *(continued)*

Trait	Method	SNP_ID	Linkage group	Position (cM)	LOD score	Minor allele frequency
SCUF	GAPIT	S519_133592	10	157.79	3.05	0.09
	MLMM	S2868_13888	10	153.21	3.16	0.2
		S99_326430	3	46.33	3.1	0.41
	MRMLM	S27_149532	5	85.27	3.18	0.41
	GAPIT, MRMLM	S1378_62536	2	40.44	3.68, 3.81	0.1
		S929_92875	2	106	3.12, 3.05	0.1
		S1142_25488	10	178.53	3.14, 3.26	0.1
	GAPIT, MLMM, MRMLM	S29076_1553	10	184.92	3.88, 4.28, 4.16	0.2
		S1050_20712	10	188.59	3.77, 3.62, 4.22	0.1
		S2275_1644	10	188.63	3.55, 3.33, 3.86	0.08
		S128_2463	4	29.6	4.33, 3.72, 4.77	0.11
JUIC	GAPIT, MRMLM	S210_30053	5	289.97	3.54, 3.79	0.18
	GAPIT, MRMLM	S210_30058	5	289.58	3.11, 3.28	0.19
	MLMM, MRMLM	S2536_21208	7	208.6	3.05, 3.14	0.14
	GAPIT, MLMM, MRMLM	S210_30038	5	288.39	3.21, 3.53, 3.32	0.18
		S210_30069	5	289.97	3.54, 3.53, 3.20	0.18
		S25_140518	6	60.61	3.44, 3.82, 3.92	0.4
		S90462_626	9	97.83	3.27, 3.28, 3.67	0.06
RUSS	GAPIT	S150_272459	9	123.17	3.77	0.36
	MLMM	S99_370238	3	32.83	3.12	0.22
		S839_1081	4	185.96	3.27	0.44
	MLMM, MRMLM	S2558_15304	4	262.4	3.16, 3.23	0.36
		S1383_61402	16	12.23	3.34, 3.10	0.37
		S213_255719	17	19.38	3.43, 3.47	0.46
		S63_393328	17	210.12	3.40, 3.24	0.35
SHAP	GAPIT	S4855_830	11	26.23	3.24	0.17
	MLMM	S64_173643	11	142.77	3.24	0.37
	GAPIT, MLMM, MRMLM	S4855_850	11	16.65	4.21, 3.69, 3.65	0.16
		S31850_361	15	79.98	3.77, 3.40, 3.69	0.08
		S31850_365	15	79.98	3.25, 3.11, 3.09	0.08
		S13375_2092	15	115.1	3.67, 3.77, 4.04	0.3
SWET	MLMM	S19_193115	13	130.74	3.2	0.32
		S19_193117	13	136.64	3.08	0.34
	MLMM, MRMLM	S3_522074	13	82.64	3.46, 3.10	0.34
	GAPIT, MLMM, MRMLM	S161_122829	4	239.02	3.35, 3.45, 3.35	0.25
		S673_155500	4	257.34	3.33, 3.67, 3.29	0.3
		S673_155490	4	257.59	3.33, 3.64, 3.29	0.3
		S182_250115	5	189.89	4.45, 4.51, 4.87	0.21
		S3_522046	13	82.81	3.11, 3.63, 3.28	0.34
		S3_522033	13	145.18	3.20, 3.68, 3.25	0.5

Supplementary Table S1 *(continued)*

Trait	Method	SNP_ID	Linkage group	Position (cM)	LOD score	Minor allele frequency
SOUR	GAPIT	S19231_632	1	150.03	3.05	0.22
		S73_183403	1	159.29	3.25	0.12
	MLMM	S3104_13218	3	74.11	3.63	0.41
		S48_62828	6	29.4	3.13	0.25
		S302_12660	6	50.36	3.36	0.48
		S719_37113	14	59.15	3.14	0.39
		S5000_3120	14	53.96	3.09	0.4
	MRMLM	S2349_45490	1	163.25	3.26	0.19
	MLMM, MRMLM	S1587_73096	3	70.36	3.49, 3.64	0.35
		S235_63703	6	78.45	3.50, 3.24	0.06
		S1352_60946	6	106.05	3.79, 3.84	0.06
		S1627_56897	6	105.2	3.75, 3.31	0.42
	GAPIT, MLMM	S2096_36898	13	69.91	3.31, 3.21	0.2
	GAPIT, MRMLM	S1430_17611	13	55.53	3.77, 3.22	0.12
		S377_162286	1	108.18	3.24, 3.17	0.16
		S2349_45611	1	163.25	3.37, 3.56	0.19
		S801_5472	6	102.16	3.13, 3.82	0.07
	GAPIT, MLMM, MRMLM	S73_183414	1	158.78	3.52, 3.98, 3.79	0.32
		S465_110990	1	187.4	4.42, 5.01, 4.92	0.33
		S1402_39914	6	109.94	3.92, 4.56, 4.41	0.49
		S41216_512	6	201.79	4.06, 4.35, 4.73	0.07
		S499_187425	6	208.68	3.93, 4.64, 4.73	0.07
		S409_219105	13	41.35	3.93, 4.43, 4.08	0.45
